# Pyometra Presenting as a Right-Lower-Quadrant Abdominal Mass With Right-Sided Bowel Dilatation on Abdominal Radiography: A Case Report

**DOI:** 10.7759/cureus.102831

**Published:** 2026-02-02

**Authors:** Seena Sugathan, Matthew D Parkinson, Syed O Husain, Bushra Jamil

**Affiliations:** 1 Acute Medicine, North Manchester General Hospital, Manchester, GBR; 2 Medicine, North Manchester General Hospital, Manchester, GBR; 3 General Medicine, Manchester University NHS Foundation Trust, Manchester, GBR

**Keywords:** abdominal radiography, intestinal obstruction, postmenopausal woman, pyometra, right-lower-quadrant mass

## Abstract

Pyometra is an uncommon uterine infection that predominantly affects postmenopausal females. It can present with non-specific features and may at times mimic gastrointestinal pathology, delaying diagnosis. An 81-year-old female with multiple comorbidities and frailty presented to the emergency department with two weeks of poor oral intake and reduced mobility. She was treated as having sepsis of unclear source with suspected respiratory infection. Despite broad-spectrum intravenous antibiotics, she deteriorated clinically with persistently raised inflammatory markers. She developed abdominal discomfort, constipation, and then abdominal distension and right-sided abdominal tenderness. An abdominal X-ray showed dilated bowel loops on the right. Pelvic magnetic resonance imaging (MRI) demonstrated a markedly distended uterine cavity containing a large fluid collection with an air-fluid level and restricted diffusion, consistent with pyometra. Malignancy could not be excluded on imaging. The case was discussed with gynaecology and microbiology, and antibiotics were escalated. A best interests meeting concluded that definitive management with uterine drainage was too invasive given her frailty and unlikely tolerance. She was managed conservatively with intravenous antibiotics, with transient improvement in delirium and inflammatory markers. This case highlights pyometra as an important differential diagnosis in elderly women presenting with abdominal symptoms and bowel dilatation on imaging. Early pelvic imaging should be considered when sepsis persists without a clear source.

## Introduction

Pyometra is a uterine infection in which pus collects inside the uterine cavity. It most often affects postmenopausal patients and commonly occurs when cervical stenosis prevents normal drainage [1]. Pyometra is uncommon; however, in postmenopausal patients, spontaneous pyometra has a meaningful association with gynecologic malignancy. Because of this, clinicians should evaluate for endometrial and cervical cancer when pyometra is diagnosed [2]. The vast majority of cases occur in postmenopausal women after the sixth decade, with a mean age of approximately 72 years [3].

Idiopathic pyometra mainly affects older, medically complex patients. Symptoms range from mild to severe. Abdominal pain and vaginal discharge are common, and some patients present with fever or sepsis [4]. In the literature, the classically described presentation includes fever, abdominal pain, and vaginal discharge or vaginal bleeding, but it is noted that clinical presentation may vary. It has also been reported in medical literature that more than half the cases of women with unperforated pyometra may be asymptomatic [5]. Cultures often grow mixed flora, with *Escherichia coli *and anaerobes such as *Bacteroides fragilis* reported among the most common isolates [6]. 

Pyometra can lead to serious complications, including septic shock and uterine perforation with diffuse peritonitis. When perforation occurs, the presentation can mimic gastrointestinal emergencies such as appendicitis or bowel perforation. Treatment depends on severity and complications. Options include antibiotics, cervical dilatation with uterine drainage, and surgery when drainage is not feasible or when perforation is present [4,6].

## Case presentation

An 81-year-old female, with past medical history significant but not limited to diabetes mellitus type 2 (DMT2), vascular dementia, aortic stenosis (AS), colonic polyp, and dyslipidaemia, presented to the emergency department (ED) after two weeks of poor oral intake and a noticeable decline from her usual mobility level. She also developed hypoactive delirium on a background of vascular dementia. Upon initial exam, she was found to be clinically dehydrated, exhibited crepitations in the bilateral lower lung bases, a systolic ejection murmur, and a white rash noted in the groin. Her initial lab workup showed raised inflammatory markers (Table 1).

**Table 1:** Inflammatory markers during the initial period of admission

Parameter	Day 1 value	Day 3 value	Day 12 value	Reference range
White blood cells	20.0x10^9^/L	25.7x10^9^/L	17.3x10^9^/L	4.0-11.0 x 10^9^/L
Neutrophils	16.86x10^9^/L	23.44x10^9^/L	14.32x10^9^/L	1.8–7.5 x 10^9^/L
C-reactive protein	103 mg/L	131 mg/L	135 mg/L	3-5 mg/L

A chest X-ray showed haziness in the right lung lower zone. A computerised tomography (CT) scan of the head did not demonstrate any acute intracranial pathology to explain the confusion. The patient was treated for sepsis of unknown source, with a likely pulmonary cause being the source. The patient was treated with amoxicillin/clavulanic acid. The suspected groin rash was treated as a suspected fungal infection. By the third day, the antibiotics were escalated to piperacillin/tazobactam due to clinical worsening. By day 12 of inpatient stay, the patient did not improve clinically and little response to continued intravenous antibiotics was seen (Table 1).

**Figure 1:**
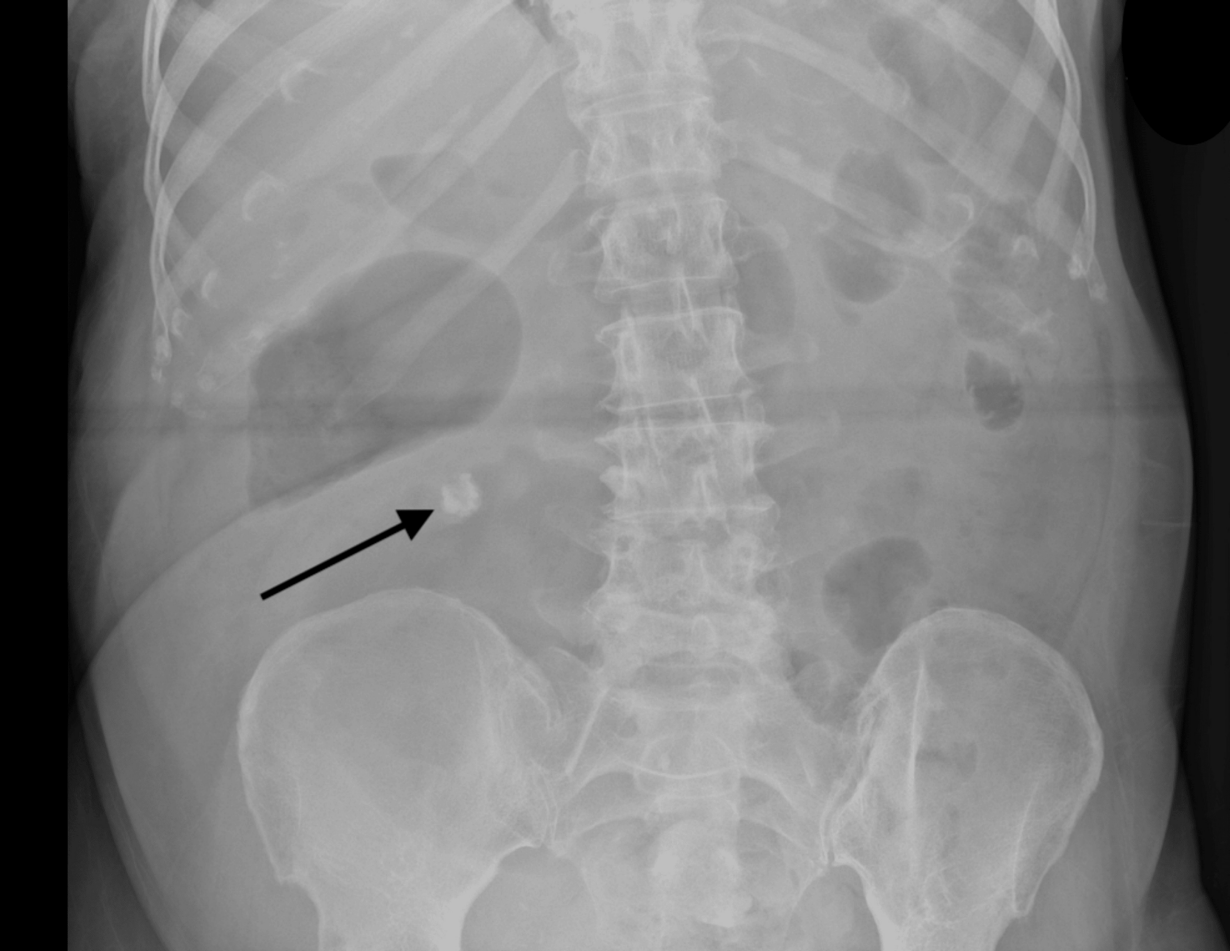
X-ray abdomen Nonspecific bowel gas pattern. Irregular calcified object at the right para-lumbar region may represent a calcified lymph node.

During her inpatient stay, the patient continued to have poor oral intake and no bowel output. On day 12, an abdominal X-ray was performed due to constipation and abdominal discomfort, which revealed dilated loops of bowel and a suspected calcified lymph node (Figure 1).

**Figure 2:**
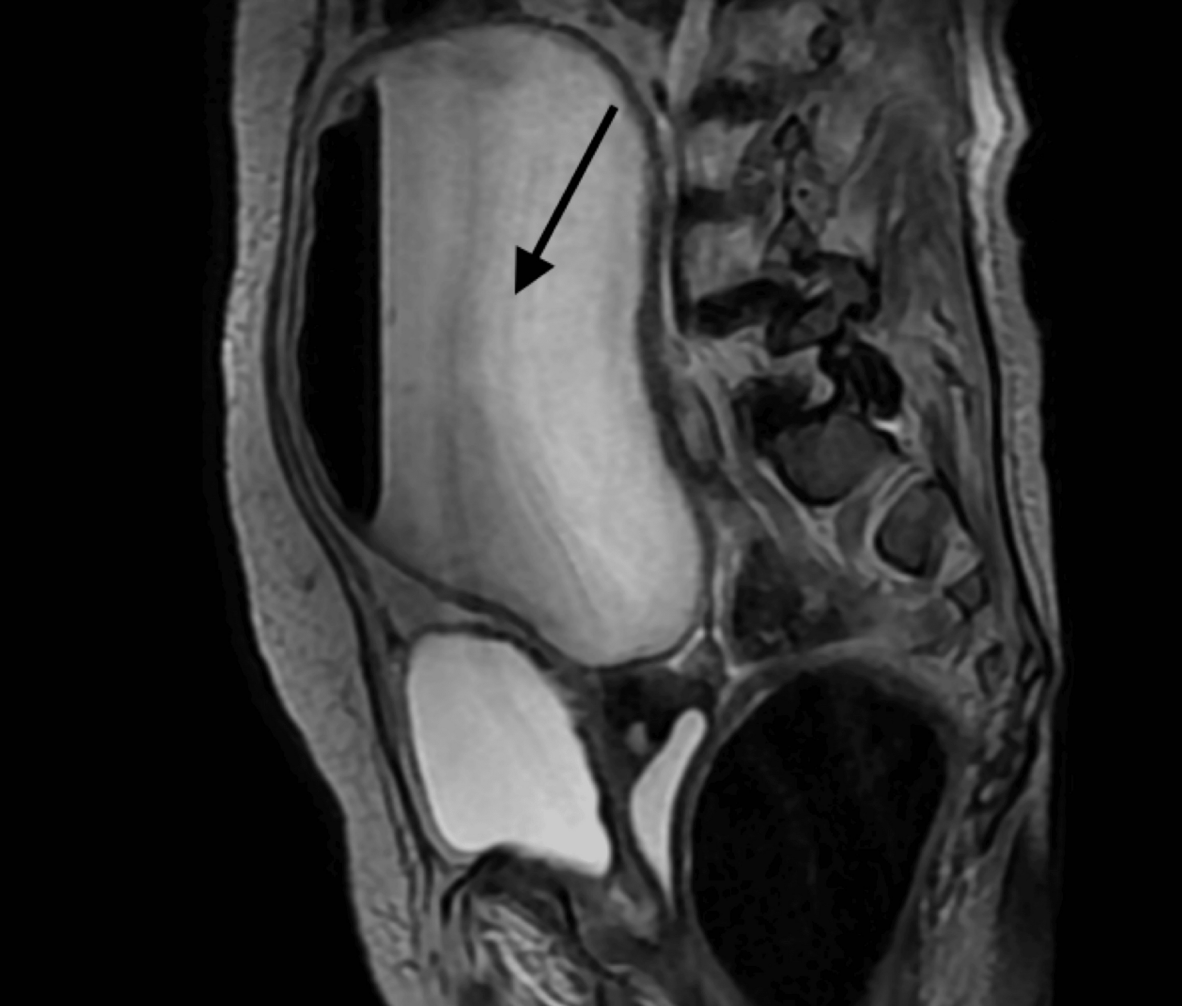
MRI pelvis The uterine cavity was seen filled with a large amount of fluid forming an air-fluid level that has shown restricted diffusion, which is suggestive of pus/pyometrium.

The patient went on to develop abdominal distension and tenderness to palpation over the right iliac fossa, right hypochondrium and epigastric regions. The MRI pelvis (Figure 2) revealed a large collection of fluid, suggestive of pyometrium, which could not rule out any malignancy, which was discussed with the gynaecology and microbiology teams. The antibiotic escalation was done upon the advice of gynaecology and microbiology.

A best interests meeting was held with the family and the multidisciplinary team. The team agreed that definitive management of pyometra would require uterine drainage, which was considered too invasive given her frailty and thus would not be tolerated. She was therefore managed conservatively with intravenous antibiotics, which resulted in a transient improvement in hypoactive delirium and inflammatory markers. By the third week of hospital stay patient had a stroke, which was complicated by a seizure, following which a meeting was held with the palliative team. Given the patient's age, frailty and prognosis, an end-of-life protocol was initiated, and the patient was placed on comfort measures. She died 3 days after placement on end-of-life measures. 

## Discussion

Pyometra, an uncommon condition characterised by the accumulation of pus within the uterine cavity, predominantly affects elderly postmenopausal women and accounts for less than 0.5% of gynaecological admissions [7-8]. It typically arises due to impaired drainage of uterine secretions, most often secondary to cervical stenosis caused by malignancy, radiation therapy, surgical scarring, or atrophic changes [2,4]. The classic clinical triad includes lower abdominal pain, purulent vaginal discharge, and postmenopausal bleeding, but many patients may remain asymptomatic or present with non-specific symptoms, delaying diagnosis [7-8]. 

In rare cases, pyometra may present as an abdominal or pelvic mass, raising concerns for malignancy or adnexal pathology. Imaging such as ultrasonography or CT scans may show an enlarged uterus with internal fluid collection, yet such findings are non-specific and may mimic other conditions such as tubo-ovarian abscesses, degenerated fibroids, or ovarian tumours [9]. 

Treatment modalities include drainage and antibiotic therapy, with the most common antibiotic regimen prescribed being amoxicillin/clavulanic acid, although there has been no reported significant difference in efficacy with metronidazole or cefuroxime [10]. Drainage, however, is suggested as first-line treatment to prevent perforation and intra-abdominal complications.

Spontaneous perforation of pyometra, although rare, is a well-documented and life-threatening complication, leading to peritonitis and sepsis if not promptly managed [7]. There are limited reports in the literature of pyometra masquerading as a large abdominopelvic mass, with most diagnoses made intraoperatively during exploratory surgery for suspected malignancy [9,11]. 

This case underscores the importance of considering pyometra in the differential diagnosis of abdominal masses in elderly women, particularly in the absence of classic infectious or gynaecological symptoms. Early recognition and appropriate intervention can prevent serious complications.

## Conclusions

Pyometra can present without classic gynaecological symptoms and may look like bowel pathology on initial imaging. In older postmenopausal patients with persistent sepsis and new abdominal findings, this pelvic pathology should be actively considered. In addition, abdominal radiography may show secondary bowel dilatation, but pelvic imaging per radiology protocol is needed to identify the uterine source. Management should balance definitive treatment with patient frailty and goals of care. Early recognition can guide specialist input, reduce delays, and help prevent severe complications such as perforation and peritonitis.
